# Lung cancer-derived exosomal miR-132-3p contributed to interstitial lung disease development

**DOI:** 10.1186/s12957-023-03095-6

**Published:** 2023-07-15

**Authors:** Sufang Fang, Ting Wang, Ling Weng, Ximei Han, Rongshan Zheng, Hongying Zhang

**Affiliations:** Respiratory Department, Fuzhou Pulmonary Hospital of Fujian Province, the Teaching Hospital of Fujian Medical University, Fuzhou, 350008 China

**Keywords:** Interstitial lung diseases, miR-132-3p, Exosome, NHLF, SPRY1

## Abstract

**Purpose:**

Interstitial lung diseases (ILDs) have high morbidity and mortality and poor prognosis. The significance of microRNAs (miRNAs) was highlighted in ILDs development. Currently, we attempted to confirm the functions of lung cancer-derived exosomal miR-132-3p and reveal the underlying mechanism.

**Method:**

Characteristics of exosomes were verified by transmission electron microscope (TEM), nanoparticle tracking analysis, and Western blot assay. Exosome uptake for the normal human lung fibroblasts (NHLF) was assessed using a PKH67 staining assay. MTT and colony formation assays were applied to examine the proliferation abilities of NHLF. The interaction between miR-132-3p and sprouty1 (SPRY1) was confirmed by a luciferase reporter assay.

**Results:**

Lung cancer-derived exosomes promoted normal human lung fibroblast activation. Exosome inhibitor GW4869 reversed the effects of Exo on NHLF. Subsequently, miR-132-3p in lung cancer-derived exosomes activated the normal human lung fibroblast and promoted interstitial lung disease development ex vivo. Next, SPRY1 was verified to be the binding protein of miR-132-3p, and sh-SPRY1 abrogated the effects of the miR-132-3p inhibitor on NHLF.

**Conclusion:**

Exosomal miR-132-3p from A549 cells accelerated the development of interstitial lung disease through binding to SPRY1, which might serve as an important target for ILDs.

**Supplementary Information:**

The online version contains supplementary material available at 10.1186/s12957-023-03095-6.

## Introduction

Interstitial lung diseases (ILDs), diffuse parenchymal lung disorders, have high morbidity and mortality, which are characterized by progressive and irreversible lung destruction including inflammation, fibrosis, and architectural distortion [1, 2]. The molecular mechanisms involved in the ILDs are poorly understood.

MicroRNAs (miRNAs) are ~ 20 *nt* small noncoding RNAs and have significant regulatory functions in interstitial lung disease development. For instance, in ILDs associated with polymyositis/dermatomyositis, miR-1 in serum was a promising novel biomarker [3]. In systemic sclerosis-associated ILDs, miR-320a mediated the collagen expression levels to regulate the fibrotic process [4]. MiR-140 overexpression downregulated osteoglycin, a potential and therapeutic target for ILDs, to suppress ILDs development via the Wnt signaling pathway [5]. MiR-7 participated in activating lung fibroblast in polymyositis-related interstitial lung diseases [6]. It was reported that miR-200c could be a biomarker of the severity of ILD because the expression levels of miR-200c had a positive correlation with the severity of ILDs [7]. MicroRNA-155 antagomir was a therapeutic miRNA to relieve the pulmonary fibrosis induced by bleomycin [8]. The inhibition of miR-34a effectively prevented pulmonary fibrosis [9]. Here, miR-7 had a significant decrease in fibrotic lung tissues, and miR-7 overexpression relieved lung fibrosis in vivo [10]. MiR-86-5p was proven to be an anti-fibrotic factor in pulmonary fibrosis [11]. These results revealed that miRNAs had significant regulatory functions in interstitial lung disease development.

The exosomes are a kind of membrane vesicles with diameters ranging from 30 to 150 nm [12]. The functional mRNAs, miRNAs, and proteins enclosed in exosomes, the effective and stable carriers, could be delivered to the microenvironment to participate in intercellular communication and exert numerous physiological functions. According to previous research, exosomes were reported to involve in the development of ILDs [13, 14].

We have boldly hypothesized that a novel therapeutic miRNA contained in exosomes from A549 cells can be used in the occurrence of interstitial lung diseases, revealing the potential mechanism of this miRNA in qualitative lung diseases and possibly providing new target support for the diagnosis and treatment of ILDs.

## Materials and methods

### Cell purchase, culture, and transfection

A549 cells and NHLF were purchased from the Chinese Academy of Science cell bank. A549 cells were cultured in RPMI-1640 medium (31,870,082, Gibco, CA, USA). NHLF were grown in DMEM (11,995,065, Gibco, CA, USA). All cultures were supplemented with 10% fetal bovine serum (FBS, 10100147C, Gibco, CA, USA) and 1% penicillin–streptomycin (15,140,122, Gibco, CA, USA). After the confluence of NHLF reached 70–80%, miR-NC, miR-132-3p mimic, and miR-132-3p inhibitor were synthesized by Ningbo Kangbeibio Biotech Ltd. and were transfected into NHLF using Lipo6000™ Transfection Reagent (C0526, Beyotime Biotechnology, Shanghai, China). The sequences such as miR-132-3p inhibitor/mimics in this study were listed: miR-132-3p inhibitor (5′-CGACCAUGGCUGUAGACUGUUA-3′), miR-132-3p mimics (sense: 5′-UAACAGUCUACAGCCAUGGUCG-3,′ and antisense: 5′-CGACCAUGGCUGUAGACUGUUA-3′).

### The isolation and purification for exosomes from A549 cells

The exosomes were isolated using Exosome Isolation Kit (UR52121; Umibio Science and Technology Group, Shanghai, China). Briefly, 20-ml supernatant of A549 cells culture was centrifuged for 20 min at 3000 g to remove the cell debris. Then, 5 ml of the exosome concentration solution was resuspended from the supernatant and incubated for 2.5 h at 4 °C. The mixture was centrifuged at 12,000 g for 60 min, resuspended in 100-μl PBS, and subsequently loaded into the exosomes purification filter to obtain the purified exosomes.

### TEM identification and NTA for the exosomes from A549 cells

TEM was used for the morphology identification of the exosomes from A549 cells. Specifically, the exosome pellets were fixed by 2.5% glutaraldehyde, dehydrated with increasing alcohol concentrations, and observed under transmission electron microscopy (JEM-1400Flash, JEOL, Tokyo, Japan). A total of 100-μl exosomes were applied to ZetaView (Malvern Panalytical) to analyze the particle size.

### PKH67 staining assay

To observe the exosome uptake for NHLF, the exosomes from A549 cells were labeled with PKH67-by-PKH67 Green Fluorescent Cell Linker Mini Kit (MINI67, Sigma, MO, USA) and were co-cultured with NHLF for 30 min. NHLF were incubated with Hoechst 33,342 staining solution (C1029, Beyotime Biotechnology, Shanghai, China) at 37 °C to obverse the nuclei. The fluorescence images were captured by the confocal microscope (Leica, Wetzlar, Germany).

### MTT assay

MTT Cell Proliferation and Cytotoxicity Assay Kit (C0009S, Beyotime Biotechnology, Shanghai, China) were applied to determine the cell viability of NHLF. In brief, NHLF were plated into 96-well plates at the density of 1 × 10^3^ cells/well. Subsequently, each well of the plate was added with 10-μl MTT reagent for 4-h incubation, treated with the formazan solvent, and measured at the wavelength of 570 nm.

### Colony formation assay

A total of 100-μl NHLF suspensions (1 × 10^4^ cells/ml) in different groups were seeded into a 6-well plate, and the NHLF were cultured in DMEM containing 10% FBS for 14 days. After culture for 14 days, the cells were rinsed with PBS, fixed, and stained. The cell clusters containing > 50 cells were counted and imaged by a CX41 light microscope (Olympus, Tokyo, Japan).

### Flow cytometry assay

Flow cytometry was employed to examine the apoptosis of NHLF [15]. NHLF in different groups were stained with Annexin V-FITC/PI Cell Apoptosis Detection Kit (AC12L033; Shanghai Life iLab Biotech Ltd., Shanghai, China). NHLF were digested, centrifuged, collected, and stained with 5 μl of FITC-Annexin V and 5-μl PI for 10 min incubation under dark. Countstar Rigel S3 flow cytometer was used for analysis.

### Western blotting assay

As previous study [16], the proteins were extracted from NHLF and the exosomes from A549 cells using RIPA buffer (high) (R0010, Solarbio, Shanghai, China) on ice for 20 min and quantified by BCA Kit (PC0020; Shanghai Acmec Biochemical Co., Ltd., Shanghai, China). All antibodies were purchased from Finetest Biotech Ltd. (Wuhan, China) or Abcam (Shanghai, China): anti-CD81 (1:500; FNab10448), anti-TSG101 (1:1000; FNab10812), anti-Alix (1:1000; ab275377), anti-tubulin (1:5000; ab6160), anti-Ki67 (1:200; FNab09788), anti-PCNA (1:5000; FNab06217), anti-Bcl-2 (1:1500, ab194583), anti-Bax (1:1500, ab32503), anti-Caspase-3 (1:2000, ab2302), anti-SPRY1 (1:1000; ab111523), and anti-β-actin (1:5000; ab8226). β-actin was used to normalize protein expression levels. Afterward, the horseradish peroxidase-labeled goat anti-rabbit HRP antibody (1:5000, ab97051) and goat anti-mouse HRP antibody (1:5000, ab205719) were purchased as the secondary antibody. Finally, protein bands were developed using ECL Plus Ultra-Sensitive Kit (PH0353, Phygene Life Sciences Company, Fuzhou, China).

### Mouse models of bleomycin (BLM)-induced interstitial lung disease

Six-week-old male C57BL/6 J (B6) mice were purchased from Cavens Biogel (Suzhou, China), raised in a clean animal cabinet at 26 ℃, 12-h light/12-h dark period, and free to obtain water and full nutrition food. For the BLM group, the mice were treated with an intratracheal injection of 1 mg/ml BLM. After 10 days, the mice were treated with 100 μg exo, exo + miR-NC, or exo-miR-132-3p inhibitor via tail vein every 3 days for three times. After 25 days, mice were sacrificed, and lung tissues were separated to observe the histological changes.

### Real-time quantitative polymerase chain reaction (RT-qPCR) assay

The total RNA from NHLF and lung tissues were extracted by TsingZol Total RNA Extraction Reagent (TSP401, Tsingke, Nanjing, China) according to previous reports [15]. HiScript II 1st-Strand cDNA Synthesis Kit (+ gDNA wiper) and miRNA 1st-Strand cDNA Synthesis Kit (R212-01 and MR101-01, correspondingly) were utilized to achieve the mRNA and miRNA expressions by ChamQ Universal or miRNA SYBR Mix (Q711-02/03 and MQ101-01/02, correspondingly). These products were purchased from Vazyme (Nanjing, China). All primers were synthesized by MBL Beijing Biotech Co., Ltd., and GAPDH or U6 was used as the internal reference. The sequences of the primers were displayed in Table 1.

**Table 1:** Primers were used in this work

	**Forward (5′-3′)**	**Reverse (5′-3′)**
miR-132-3p	GCGCGTAACAGTCTACAGCCA	AGTGCAGGGTCCGAGGTATT
SPRY1	TGGATGACTTGAAGGGTT	CAAACAGGATGGTAGGGT
COL1A1	AAGGTGTTGTGCGATGACG	TGGTCGGTGGGTGACTCTG
COL3A1	GAGCTGGCTACTTCTCGC	TCTATCCGCATAGGACTGAC
α-SMA	GGGGTGATGGTGGGAATG	GCAGGGTGGGATGCTCTT
FN	TGTTATGGAGGAAGCCGAGGTT	GCAGCGGTTTGCGATGGT
CXCL12	CTGTGCCCTTCAGATTGTA	GCTTTCTCCAGGTACTCCT
IL-1β	ACAGTGGCAATGAGGATG	TGTAGTGGTGGTCGGAGA
IL-6	GGAGACTTGCCTGGTGAA	GCATTTGTGGTTGGGTCA
IL-8	CTCCAAACCTTTCCACCCC	GATTCTTGGATACCACAGAGAATG
TGF-β1	CTCGGGGGCTGCGGCTACTG	GGCGTATCAGTGGGGGTCA
GAPDH	GAAGGTGAAGGTCGGAGTC	GAAGATGGTGATGGGATTTC
U6	CTCGCTTCGGCAGCACATATA	AACGCTTCACGAATTTGCGT

### Luciferase reporter assays

To validate further whether sprouty1 (SPRY1) was a direct target of miR-132-3p, the binding sites of wild-type (WT) and mutant (MUT) sequences of SPRY1 3′UTR with miR-132-3p were cloned into the pmirGLO vector to generate SPRY1-WT or SPRY1-MUT [17]. These reporter plasmids were co-transfected with miR-132-3p mimic or mimic NC into NHLF for 48 h. The luciferase activity was assessed by the Luciferase Reporter Gene Assay Kit (RG027, Beyotime Biotechnology, Shanghai, China).

### Statistical analysis

Statistical analysis was performed using GraphPad Prism 7.0 software. Data were demonstrated as means ± standard deviation (SD). Two-tailed Student’s *t*-test and one-way ANOVA with Turkey’s test were performed to compare the difference between two or multiple groups, respectively. Data with *P*-values smaller than 0.05 was considered a significant difference.

## Result

### Characterization of isolated exosomes from A549 cells

**Fig. 1:**
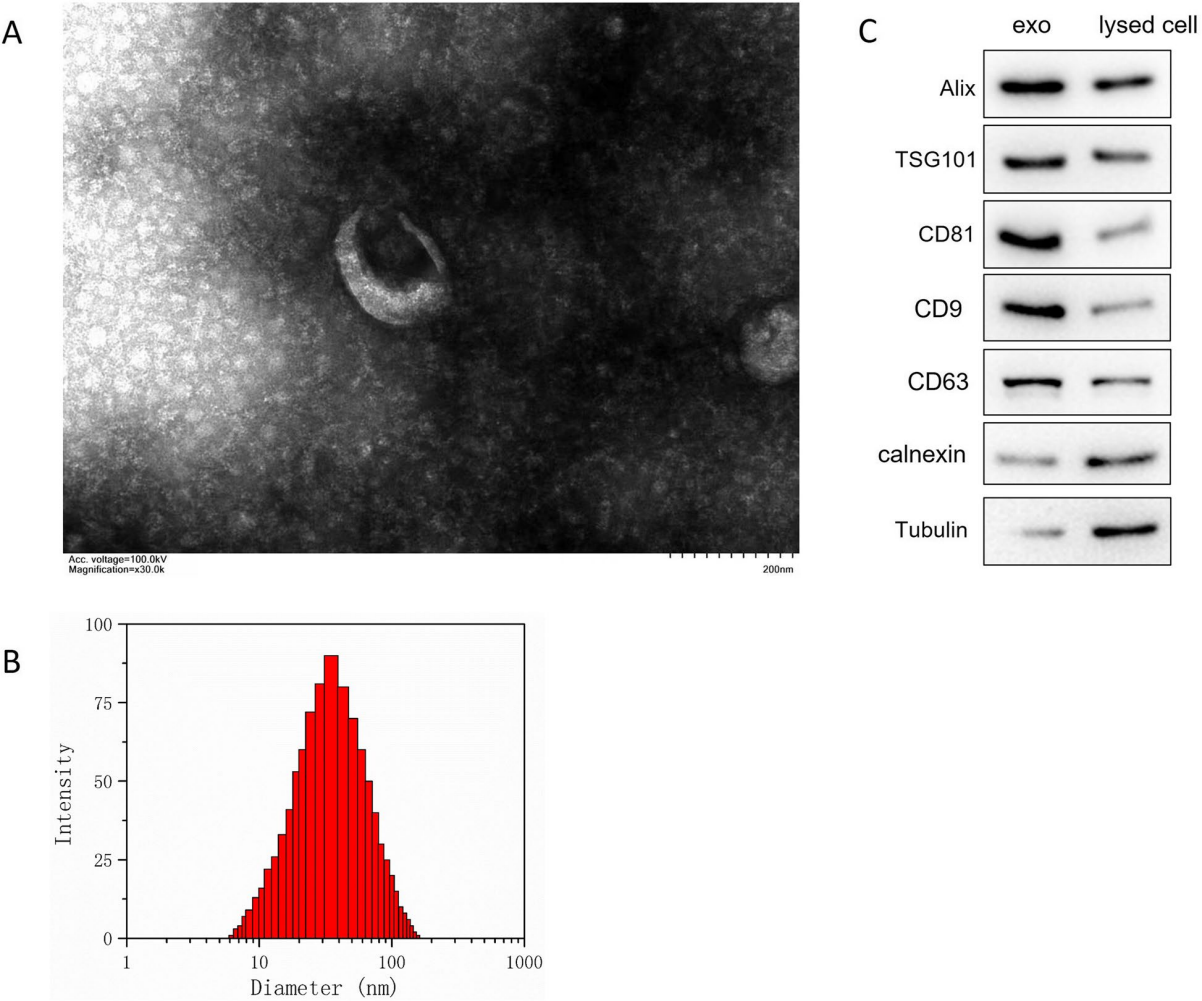
The exosomes from A549 cells were identified. **A** The structure of exosomes from A549 cells was identified by TEM. **B** The diameter for the exosomes from A549 cells was tracked by NTA. **C** The representative exosomal markers such as Alix, TSG101, CD9, CD63, calnexin and CD63, and tubulin protein expressions were detected in lung cancer-derived exosomes and corresponding cells

In Fig. 1A and B, the prepared exosomes from A549 cells were identified by TEM and NTA, which indicated that the isolated exosomes from A549 cells had a 120-nm average diameter and typical two-layer membrane structure. In Fig. 1C, the exosomal positive indicators CD9, CD63, TSG101, Alix, and CD81, and one negative indicator calnexin, were used to determine the exosomes. There was a significant increase for Alix, TSG101, CD9, CD63, and CD81 protein levels in exo group compared with the corresponding cells. The tubulin and calnexin protein were expressed in cells but were absent in exosomes, which suggested that the isolated exosomes from A549 cells were successfully prepared.

### Tumor-derived exosomes regulated normal human lung fibroblast activation

**Fig. 2:**
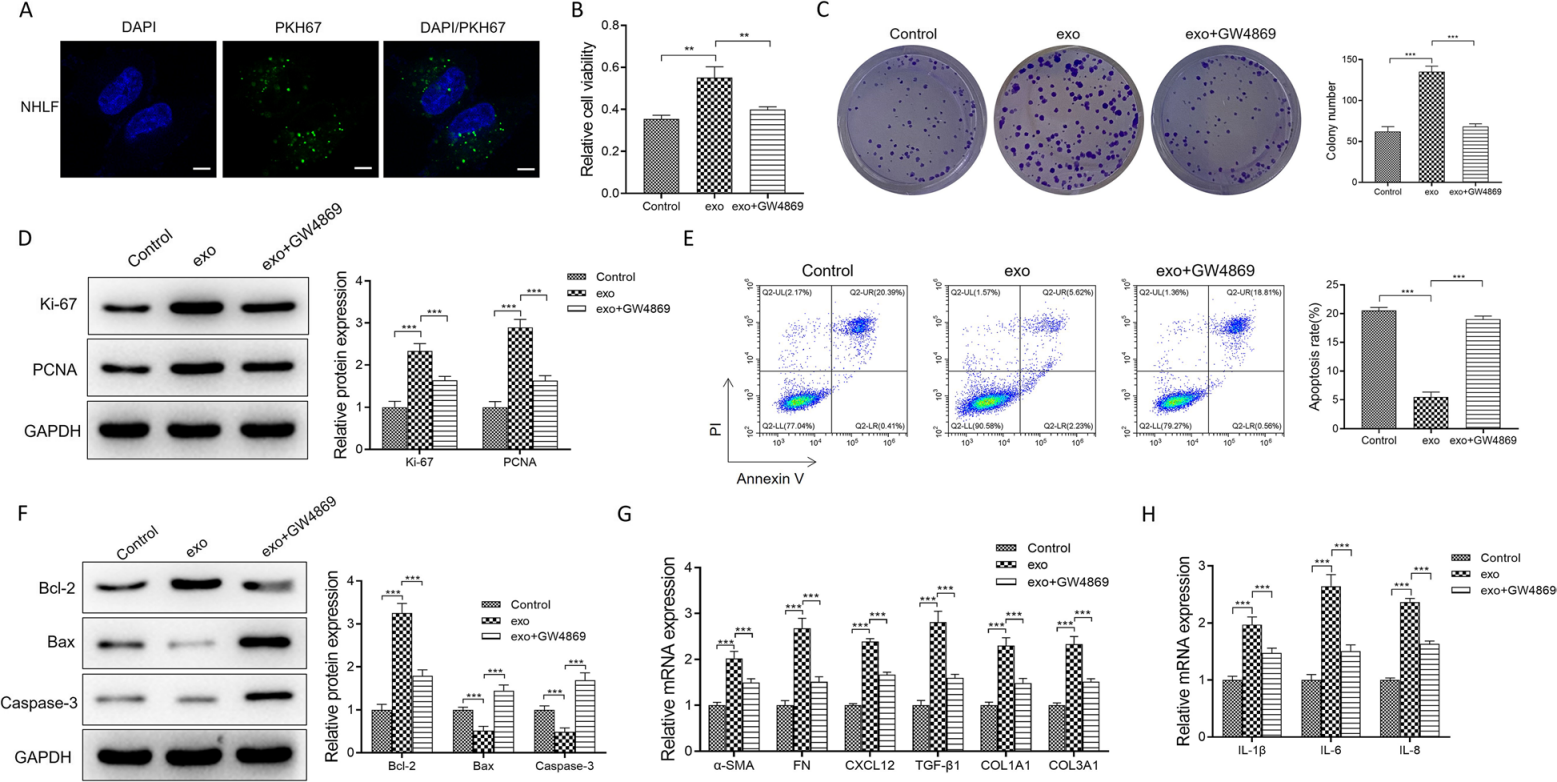
The exosomes from A549 cells activated the normal human lung fibroblast. **A** The green fluorescence levels were detected for NHLF co-cultured with PKH67-labeled exosomes from A549 cells. **B** MTT assay tested the viability of NHLF treated with exosomes or exosomes + GW4869. **C** The proliferation abilities of NHLF, which were incubated with exosomes or exosomes + GW4869, were checked by clone formation assay. **D** The expression levels of proliferation-related proteins (Ki-67 and PCNA) were tested for control, exo and exo + GW4869 group. **E** Flow cytometry assay was performed to analyze the apoptosis rates in all groups. **F** Western blot analysis of the pro-/anti-apoptosis proteins (Bax and Caspase-3 and Bcl-2) was performed. **G**–**H** α-SMA, FN, TGF-β, COL1A1, COL3A1, CXCL12, IL-1β, IL-6, and IL-8 mRNA expressions were tested by qRT-PCR analysis. Data were presented as mean ± SD of three independent experiments. ***P < 0.001; **P < 0.01

To determine the function of lung cancer-derived exosomes, the prepared exosomes from A549 cells were labeled with PKH67 and co-cultured with NHLF, the normal human lung fibroblasts. In Fig. 2A, there was the strongest green fluorescence accumulation in the cytoplasm of NHLF, suggesting that the exosomes from A549 cells could be specifically up taken by NHLF. Functionally, clone formation, MTT, and Western blot assays showed that the exosomes from A549 cells promoted the viability and proliferation ability and the proliferation-related protein levels such as ki-67 and PCNA for NHLF. However, GW4869, an exosome biogenesis inhibitor, partly reversed the promotion effect of lung cancer-derived exosomes on the viability and proliferation of NHLF in Fig. 2B–D. Next, in Fig. 2E–F, flow cytometry, and Western blot assays, revealed that NHLF co-cultured with the A549 cells-derived exosomes inhibited the apoptosis rates and the pro-apoptosis proteins (Bax and Caspase-3) expressions and elevated the expression of the anti-apoptotic protein (Bcl-2). In the markers of fibrosis-associated genes such as alpha-smooth muscle actin (α-SMA), fibronectin (FN) and transforming growth factor β (TGF-β), collagen genes such as collagen type 1 (COL1A1) and collagen type 1 (COL3A1), the homeostatic chemokine (C-X-C motif chemokine ligand 12, CXCL12), and many pro-inflammatory mediators such as IL-1β, IL-6, and IL-8, their mRNA levels were significantly upregulated in exo group compared with the control group (NHLF without any treatments) in Fig. 2G–H. The promotion effect resulting from the lung cancer-derived exosomes was weakened by GW4869 treatment.

### MiR-132-3p in exosomes mediates normal human lung fibroblast activation

**Fig. 3:**
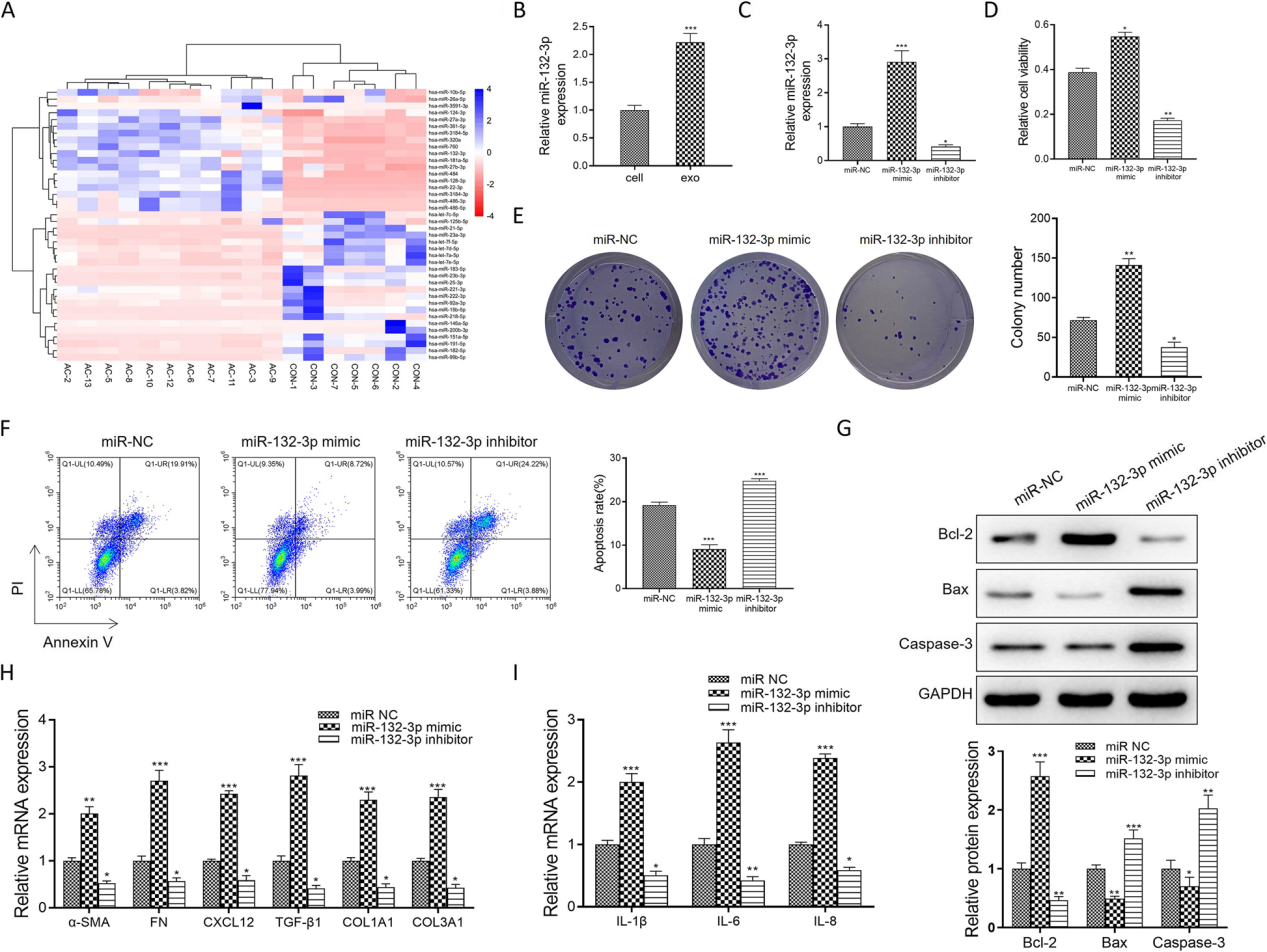
MiR-132-3p in exosomes activated the normal human lung fibroblast. **A** MiR-132-3p had a high expression in the non-small-cell lung tumor-derived exosomal miRNAs. **B** The qRT-PCR tested the expression of miR-132-3p in exosomes from A549 cells and the corresponding cells. **C** In NHLF, the transfection efficiency of miR-132-3p mimic or inhibitor was evaluated. **D** MTT assays tested the cell viability in miR-132-3p mimic, miR-132-3p inhibitor, and miR-NC groups. **E** Clone formation assays tested the cell proliferation in all groups. **F** Flow cytometry analyzed the apoptosis rates in all groups. **G** Western blot assay was performed to analyze the expression levels of Bax, Bcl-2, and cleaved caspase-3 in NHLF transfected with miR-132-3p mimic, miR-132-3p inhibitor, or miR-NC. H-I. The mRNA expressions, including α-SMA, FN, TGF-β, COL1A1, COL3A1, CXCL12, and IL-1β/6/8, were tested by qRT-PCR analysis. Data were presented as mean ± SD of three independent experiments. *P < 0.05; **P < 0.01, ***P < 0.001

MiR-132-3p was reported as the non-small-cell lung tumor-derived exosomal miRNA biomarker [18]. We chose the miR-132-3p for further research in Fig. 3A. The result of qRT-PCR in Fig. 3B showed that miR-132-3p had a significantly high expression in exosomes from A549 cells compared with corresponding cells. To investigate the biological function of miR-132-3p, miR-132-3p mimic, miR-132-3p inhibitor, and miR-NC were transfected into NHLF. In Fig. 3C, the qRT-PCR results showed that the relative miR-132-3p expression was upregulated by miR-132-3p mimic. And there was an opposite trend in the miR-132-3p inhibitor group. Functionally, in Fig. 3D and E, MTT and clone formation assays showed that miR-132-3p mimic promoted the cell viability and proliferation of NHLF, whereas these phenotypes for NHLF transfected with miR-132-3p inhibitor were greatly inhibited. Next, flow cytometry assay revealed that the apoptosis rates of NHLF transfected with miR-132-3p mimic were decreased compared with miR-NC; however, in Fig. 3F, the miR-132-3p inhibitor group had a high apoptosis rate for NHLF. In Fig. 3G, miR-132-3p overexpression resulted in the downregulated expression of pro-apoptosis proteins and elevated levels of anti-apoptotic protein. In Fig. 3H and I, the miR-132-3p overexpression group had a high expression of α-SMA, FN, TGF-β, COL1A1, COL3A1, CXCL12, IL-1β, IL-6, and IL-8; however, miR-132-3p inhibitor brought about opposite effects.

### MiR-132-3p in exosomes promoted interstitial lung disease developmentin vivo

**Fig. 4:**
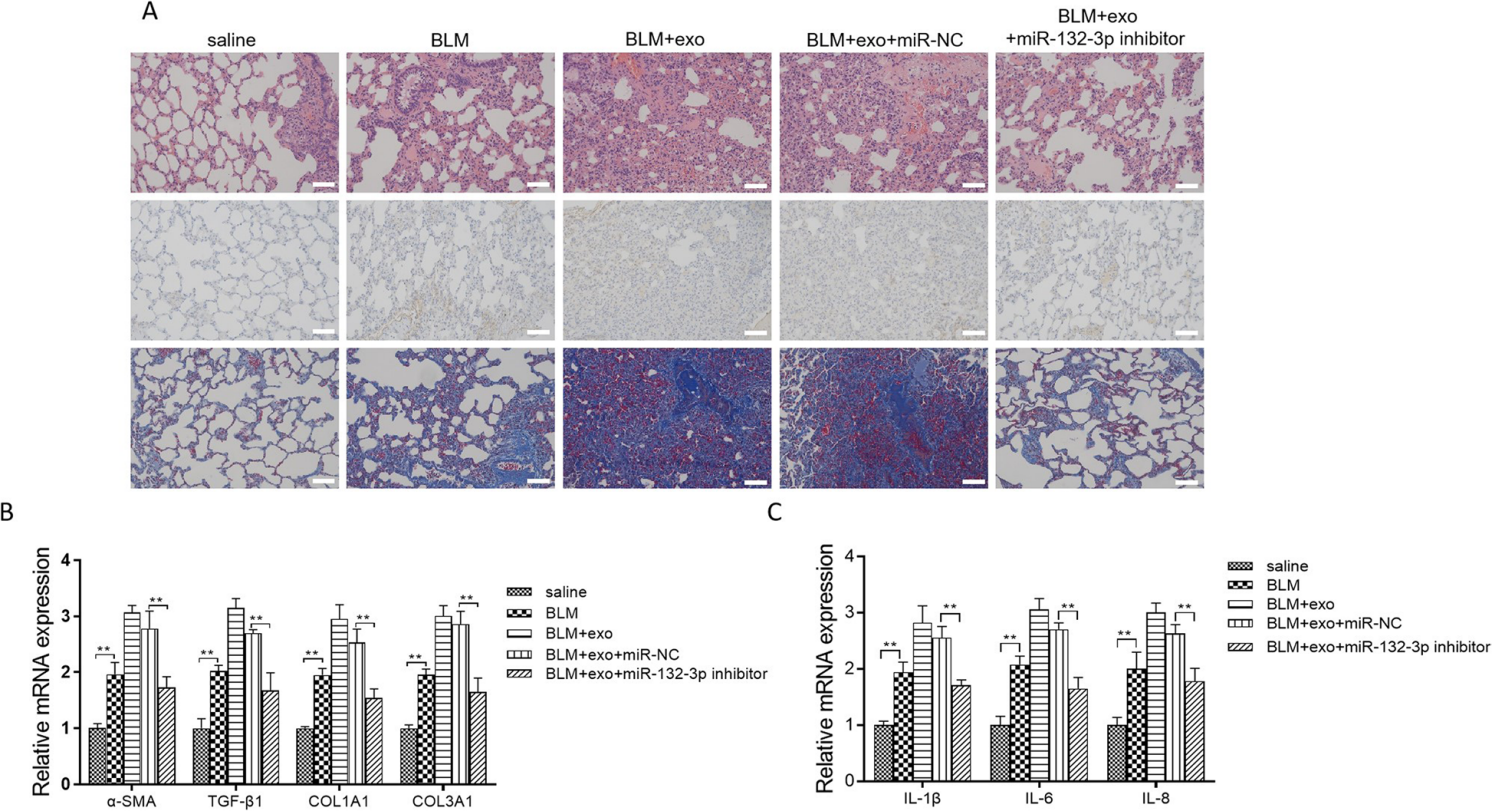
MiR-132-3p in exosomes aggravated interstitial lung disease development in vivo. **A** Representative histological lung sections were subjected to H&E and Masson staining and immunohistochemical analysis for α-SMA from saline, BLM, BLM + Exo, BLM + miR-NC-exo, and BLM + miR-132-3p inhibitor-exo groups. **B** In all groups, α-SMA, COL1A1, COL3A1, and TGF-β1 mRNA expressions were tested by qRT-PCR analysis. **C** In saline, BLM, BLM + Exo, BLM + miR-NC-exo, and BLM + miR-132-3p inhibitor-exo groups, the mRNA expressions of IL-1β/6/8 were tested. Data were presented as mean ± SD of three independent experiments. ***P < 0.001; **P < 0.01

To further explore the underlying mechanism of exosomal miR-132-3p in interstitial lung disease development, exo, miR-NC-exo, and miR-132-3p inhibitor-exo from A549 cells were used to treat the bleomycin (BLM)-induced fibrosing lung injury. In Fig. 4A, Masson’s trichrome, H&E staining, and IHC results showed that BLM-treated mice developed pulmonary fibrosis and had a high expression of α-SMA compared to the saline group. The degrees of pulmonary fibrosis in the exo and miR-NC-exo groups were markedly greater than in the BLM group. Interestingly, the treatment with miR-132-3p inhibitor-exo significantly inhibited the alveolar wall thickening, infiltration of inflammatory cells into the interstitium, and reduction in collagen deposition induced by BLM. In Fig. 4B, the fibrosis-associated genes (α-SMA and TGF-β), and collagen genes (COL1A1 and COL3A1) in BLM + exo and BLM + miR-NC-exo, had a remarkable upregulation compared with saline or BLM group. However, miR-132-3p inhibitor-exo effectively reduced the α-SMA, COL1A1, COL3A1, and TGF-β1 mRNA levels. Moreover, in Fig. 4C, the mRNA expression of IL-1β/6/8 was consistent with the fibrosis/collagen-associated genes.

### Sprouty1 (SPRY1) may be a target of miR-132-3p

**Fig. 5:**
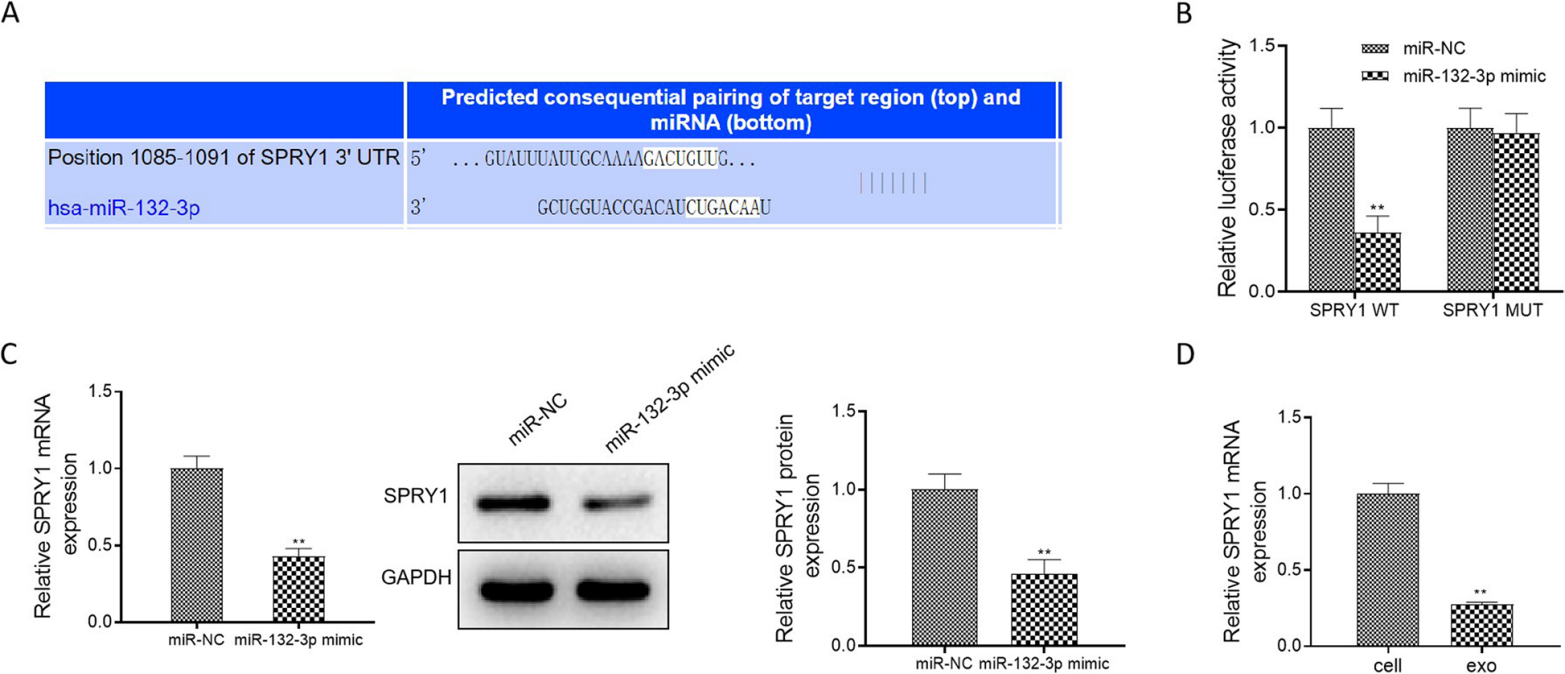
miR-132-3p interacted with SPRY1. **A** Bioinformatic analysis of the predicted interaction sites between miR-132-3p and SPRY1 was performed by TargetScan analysis. **B** Luciferase reporter assay was used to assess the relationship between miR-132-3p and SPRY1. **C** The mRNA and protein levels of SPRY1 were assessed. **D** In the isolated exosomes and A549 cells, the mRNA of SPRY1 was assessed. Data were presented as mean ± SD of three independent experiments. **P < 0.01

We searched the potential binding genes of miR-132-3p in the TargetScan database and found that SPRY1 may be a target of miR-132-3p in Fig. 5A. In Fig. 5B, luciferase assay displayed that the luciferase activity of SPRY1-WT was diminished in response to miR-132-3p mimic, while there was no significant difference in the SPRY1-MUT group, indicating that SPRY1 was the target gene for miR-132-3p. In Fig. 5C, miR-132-3p overexpression inhibited the mRNA and protein levels of SPRY1. In addition, the mRNA of SPRY1 in the isolated exosomes had a lower expression compared with A549 cells (Fig. 5D).

### Exo-miR-132-3p regulated the development of interstitial lung disease by targeting SPRY1 in NHLF

**Fig. 6:**
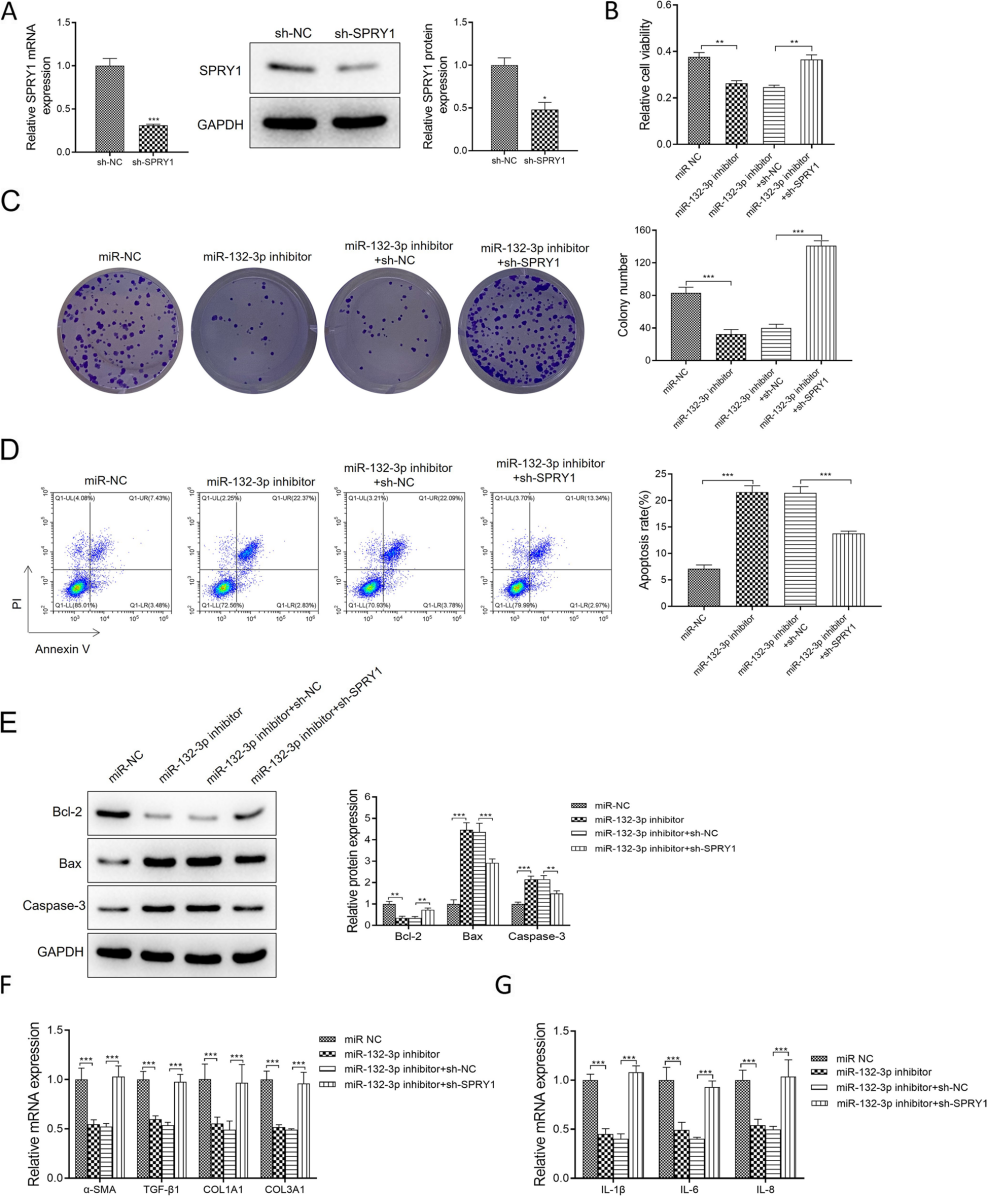
Inhibition of miR-132-3p abrogated the progression of interstitial lung disease via targeting SPRY1 in NHLF. **A** The knockdown efficiency of SPRY1 in NHLF was tested by qRT-PCR and Western blot assays. **B** Cell viabilities were assessed in NHLF transfected with miR-NC, miR-132-3p inhibitor, miR-132-3p inhibitor + sh-NC, and miR-132-3p inhibitor + sh-SPRY1. **C** Cell colony numbers were detected in all groups. **D** Flow cytometry analyzed the apoptosis rate in all groups. **E** The anti-/pro-apoptosis protein expressions were assessed by Western blot assay. **F-G** The fibrosis/collagen-associated genes (α-SMA, TGF-β1, COL1A1, and COL3A1), and many pro-inflammatory mediators (IL-1β/6/8) mRNA levels, were tested by qRT-PCR assay. Data were presented as mean ± SD of three independent experiments. ***P < 0.001; **P < 0.01

We further examined the functional correlation between SPRY1 and exosomal miR-132-3p from A549 cells. The results in Fig. 6A showed that the mRNA and protein expression of SPRY1 markedly decreased in sh-SPRY1 group. Cell viability of NHLF markedly decreased by the inhibition of miR-132-3p, but knockdown of SPRY1 reversed the decrement induced by miR-132-3p inhibitor in Fig. 6B. As expected, the cell colony number of NHLF exhibited a similar variation trend with cell viability in Fig. 6C, whereas cell apoptosis rates and pro-apoptosis proteins showed the reverse results in Fig. 6D–E. In Fig. 6F–G, the knockdown of SPRY1 significantly reversed the inhibition effect of the miR-132-3p inhibitor on the mRNA levels of the fibrosis/collagen-associated genes (α-SMA, TGF-β1, COL1A1, and COL3A1) and many pro-inflammatory mediators (IL-1β/6/8). Collectively, these results demonstrated that miR-132-3p targeted SPRY1 to regulate the development of NHLF.

## Discussion

Exosomes have become an important factor to affect the progression of fibrotic diseases by transferring anti-fibrotic or pro-fibrotic miRNAs to target cells and influencing the occurrence of pathological fibrosis. The potential functions of exosomal miRNAs on the development of interstitial lung disease laid the groundwork for understanding the molecular mechanism of ILDs [19, 20]. It was reported that the exosomes effectively promoted lung repair in pulmonary fibrosis because the treatment of exosomes decreased collagen accumulation and myofibroblast proliferation [21]. Furthermore, overwhelming studies have revealed that the anti-fibrotic miR-142-3p from macrophage-derived exosomes attenuated the pulmonary fibrosis progression [19]. The exosomal let-7d and miR-16 from serum have important roles in the pathogenesis of idiopathic pulmonary fibrosis [22]. In our work, the exosomes derived from A549 cells were demonstrated to exhibit a promotive role in normal human lung fibroblast activation and pulmonary fibrosis. Based on the results of a high-throughput sequencing, unregulated miR-132-3p was screened out, and miR-132-3p overexpression aggravated the fibrotic process of normal human lung fibroblast by activating the fibrosis/pro-inflammatory-associated gene mRNA levels. This is consistent with the study of Pellegrini Kathryn L. et al., in which miR-132-3p levels are upregulated in the entire renal cortex of mice and humans in the presence of severe fibrosis [23]. In vivo, the exosomes with inhibition of miR-132-3p significantly inhibited the pulmonary fibrosis induced by BLM.

Apart from the interstitial lung disease, miR-132-3p overexpression resulted in a size enlargement of cardiac fibroblasts to mediate cardiac fibrosis [24]. In addition, miR-132 inhibitor markedly inhibited the Ang II-induced activation of cardiac fibroblasts and cardiomyocyte hypertrophy [25]. Inhibition of miR-132 reduced renal fibrosis by weakening myofibroblast proliferation [26]. MiR-132 was downregulated to inhibit the JAK-STAT signaling pathway to alleviate detrusor fibrosis [27]. The increase of miR-132 attenuated liver fibrosis by inducing the degradation of connective tissue growth factor (CTGF) mRNA [28]. In general, it implied that miR-132 served as an effector molecule in multiple fibrotic diseases.

In numerous fibrosis diseases, miR-132 was reported to have multiple targets. In atrial fibrosis, miR‑132 targeted CTGF [29]. And CTGF was a major pro‑fibrotic factor, which promotes the synthesis of extracellular matrix to accelerate the generation of fibrosis [30]. MiR-132 also targeted and inhibited the phosphatase and tensin homolog (PTEN) expression to attenuate cardiac fibrosis [31]. In our work, miR-132-3p targeted and inhibited sprouty1 (SPRY1) to promote the development of interstitial lung disease. And the knockdown of SPRY1 significantly reversed the inhibition effect of miR-132-3p inhibitor on the ILDs progress. In liver fibrosis disease, miR-21 had a positive correlation with liver fibrosis, and overexpression of miR-21 promoted collagen production and inflammasome activation when the expression of SPRY1 was inhibited [32]. In cardiac fibroblasts, the degradation of Spry1 contributed to fibrosis [33]. For atrial fibrosis, the proliferation abilities of cardiac fibroblasts and the expression of TGF-β1 were significantly promoted when Spry1 was knock down [34]. These reports suggested that the degradation of Spry1 contributed to accelerating the process of fibrosis in multiple fibrotic diseases.

## Conclusions

To sum up, exosomal miR‑132-3p from A549 cells promoted fibrosis in NHLF by downregulating the expression of Spry1. Our study took insight into the underlying mechanism of the interstitial lung disease, and proved miR‑132-3p as a potent target for ILDs, which provided a novel aspect for the treatment of ILDs.

## Supplementary Information

**Additional file 1. **Supplementary figures and information.

## Data Availability

The datasets used and/or analyzed during the current study are available from the corresponding author on reasonable request.
